# Epithelial Mesenchymal Transition and Pancreatic Tumor Initiating CD44+/EpCAM+ Cells Are Inhibited by γ-Secretase Inhibitor IX

**DOI:** 10.1371/journal.pone.0046514

**Published:** 2012-10-19

**Authors:** Vindhya Palagani, Mona El Khatib, Uta Kossatz, Przemyslaw Bozko, Martin R. Müller, Michael P. Manns, Till Krech, Nisar P. Malek, Ruben R. Plentz

**Affiliations:** 1 Department of Internal Medicine I, Medical University Hospital, Tuebingen, Germany; 2 Department of Internal Medicine II, Medical University Hospital, Tuebingen, Germany; 3 Department of Gastroenterology, Hepatology and Endocrinology, Hannover Medical School, Hannover, Germany; 4 Institute for Pathology, Hannover Medical School, Hannover, Germany; The University of Kansas Medical Center, United States of America

## Abstract

Pancreatic ductal adenocarcinoma (PDAC) is an aggressive disease with a high rate of metastasis. Recent studies have indicated that the Notch signalling pathway is important in PDAC initiation and maintenance, although the specific cell biological roles of the pathway remain to be established. Here we sought to examine this question in established pancreatic cancer cell lines using the γ-secretase inhibitor IX (GSI IX) to inactivate Notch. Based on the known roles of Notch in development and stem cell biology, we focused on effects on epithelial mesenchymal transition (EMT) and on pancreatic tumor initiating CD44+/EpCAM+ cells. We analyzed the effect of the GSI IX on growth and epithelial plasticity of human pancreatic cancer cell lines, and on the tumorigenicity of pancreatic tumor initiating CD44+/EpCAM+ cells. Notably, apoptosis was induced after GSI IX treatment and EMT markers were selectively targeted. Furthermore, under GSI IX treatment, decline in the growth of pancreatic tumor initiating CD44+/EpCAM+ cells was observed *in vitro* and in a xenograft mouse model. This study demonstrates a central role of Notch signalling pathway in pancreatic cancer pathogenesis and identifies an effective approach to inhibit selectively EMT and suppress tumorigenesis by eliminating pancreatic tumor initiating CD44+/EpCAM+ cells.

## Introduction

Pancreatic ductal adenocarcinoma (PDAC) is a leading cause of mortality and morbidity with 5-year survival rate of 6% in Europe and the US [1]. More than 85% of patients show distant metastasis at the time of diagnosis, which render them unsuitable for surgery [2]–[4]. Despite of large numbers of clinical trials with conventional and targeted therapies, current treatments only offer limited benefit [5]. Thus, strategies are still needed to overcome this deadly disease.

Pancreatic cancer is characterized by a series of highly recurrent genetic abnormalities, including activation of the KRAS oncogene and inactivation of the TP53, SMAD4, BRCA2 and CDKN2A tumor suppressor genes [6]–[9]. Though a number of molecular markers are associated with poor outcomes in pancreatic cancer, one of the important factors contributing for this malignancy is loss of epithelial differentiation. This is manifested as epithelial mesenchymal transition (EMT), which is characterized by the gain of stem cell properties, which promotes cancer invasion and metastasis [10], [11]. The hallmark of EMT is the loss of the homotypic adhesion molecule epithelial cadherin (E-cadherin) and gain of mesenchymal markers. In line with the “cadherin switch”, epithelial-specific junction protein E-cadherin is down regulated and mesenchymal proteins such as neural-cadherin (N-cadherin) are upregulated [12]. E-Cadherin expression is under the negative regulation of the Snail, Slug and Twist transcription factors that can act as master regulators of EMT [13] and may be a downstream target of activated Kras^G12D^ [14]. In addition to the loss of E-cadherin, the induction of N-cadherin itself might contribute directly to cancer metastasis [11].

Resistance to chemo- and radio-therapy in several human cancers is linked to a population of cells with stem cell properties, namely cancer stem cells (CSCs) [15]–[19]. A number of subpopulations within PDAC have been shown to have tumor initiating or CSC properties, and appear to be hierarchically organized [20]–[22]. First, it was demonstrated that CD44+, CD24+ and ESA+ (EpCAM+) positive PDAC cancer cells show stem cell properties and enhanced tumor initiating capacity compared to bulk tumor cells [23]. Similar features were shown for CD133+, Aldehyde Dehydrogenase-1+ and c-Met+ subpopulations of PDAC cells [16], [24], [25]. Pancreatic CSCs were successfully eliminated by Hedgehog and mTOR inhibitors [26].

The Notch signalling pathway is involved in the development and progression of several malignancies [27]. The interaction of Notch ligands with their receptors, promotes a γ-secretase-dependent cleavage of the Notch receptor and release of the Notch intracellular domain (NICD) resulting in activation of the pathway [27], [28]. NICD translocates to the nucleus and induces target genes like Hairy enhancer of split (Hes1). We and others have shown that Notch signalling pathway components are upregulated in murine and human PDAC and that pharmacological or genetic inhibition of Notch suppresses PDAC development in genetically engineered mouse models [24]–[35]. Notch signalling may also be important in advanced PDAC as gamma secretase inhibitors, which abrogate Notch signalling, can suppress the proliferation of human PDAC cell lines. Moreover, recent studies have shown that pancreatic CSCs express high level of Notch1 and Notch2 [36]–[38], suggesting that Notch signalling may be important in the maintenance of CSCs. Thus, inhibition of Notch may not only prevent the emergence of PDAC in experimental models, but also be an effective therapeutic approach in advanced pancreatic cancer.

Despite the active investigation in this area, the roles for Notch in pancreatic cancer cell biology remain incompletely understood. In the present study, we sought to further examine the potential benefit of targeting Notch in pancreatic cancer by studying in detail the cell biological impact of γ-secretase inhibitor IX (GSI IX) in human pancreatic cancer cell lines. Our work reveals that the GSI can selectively block epithelial-mesenchymal transition (EMT), migration and invasion in human pancreatic cancer cell lines, and can suppress pancreatic tumor initiating CD44+/EpCAM+ cells in a xenograft mouse model.These findings support the development of therapeutic strategies targeting Notch signalling in pancreatic cancer.

## Material and Methods

### Cell Culture

Human pancreatic cancer cell lines BxPC3 (cultured from a primary tumor without evidence of metastasis) and KP3 (cultured from liver metastases of a human pancreatic tumor) were obtained from the laboratory of Nabeel Bardeesy (MGH Cancer Center, Boston, USA). These cell lines were originally obtained from the MGH Center for Molecular Therapeutics (CMT), which conducts routine cell line authentication testing by SNP and STR analysis. The cell lines were maintained at 37°C under a 5% CO2 environment in RPMI 1640+L-Glutamine+25 mM HEPES (Invitrogen, Karlsruhe, Germany) enriched with 10% fetal calf serum (FCS) (Biochrom, Berlin, Germany) and antibiotics of penicillin/streptomycin (100 units/ml) (Invitrogen, Karlsruhe, Germany).

### Drug and Treatment

For the *in vitro* experiments γ-Secretase inhibitor IX (Calbiochem, EMD Chemicals Inc., Darmstadt) was prepared as a 10 mM stock in dimethyl sulfoxide, DMSO (AppliChem, Darmstadt, Germany). Cells were treated with DMSO or γ-Secretase inhibitor IX in different concentrations (2.5 µM, 5 µM, 10 µM) and were analyzed after 48 hrs and 96 hrs. Animals were treated intraperitoneally with a drug concentration of 10 mg/kg/body weight once daily or vehicle (control) for 5 weeks, using a 3 days on and 4 days off intermittent dose schedule as described previously [36].

### Determination of cell number and proliferation

A total 5×10^3^ cells were seeded per well into a ninety 96-well tissue culture plates and were incubated over night. Following the treatment with GSI in different concentration (2.5 µM, 5 µM, 10 µM), cells were further incubated for additional time points (24, 48, 72 and 96 hrs). 10 µl of WST-1 reagent (Roche Diagnostics, Mannheim, Germany) was added per well and was incubated for two hours at 37°C to assess the cell number. The plates were read at a wavelength of 492 nm with reference wavelength of 650 nm using a Micro plate-Reader Multiskan Plus(Titertek-Berthold, Pforzheim, Germany).

### Apoptosis Assays

Cells were seeded in 6-well tissue culture plates at a density of 8×10^4^ cells per well and were incubated over night at 37°C. These cells were further treated with GSI as described in proliferation assay; 96 hrs after GSI treatment, floating cells were collected and adherent cells were trypsinized, washed twice with ice-cold PBS. The cells were then resuspended in 1 ml of 1× binding buffer and were stained with Annexin V-FITC and PI according to the manufacture's instruction using Annexin V Apoptosis Detection Kit II (BD Biosciences, San Diego, USA). The signal was detected using FACS calibur flow cytometer (BD, Heidelberg, Germany) and analyzed using FlowJo Version 8.7 software (Tree Star Inc., Ashland, USA).

### Wound healing assay

Pancreatic cancer cell lines were seeded in a 6-well plate and left to reach 80% confluence. Initially, cells were starved for 24 h in media containing 2% FCS. Then KP3 and BxPC3 cell lines were further incubated for 48 h in the starvation media containing either the controls (DMSO) or GSI (Calbiochem, EMD Chemicals Inc., Darmstadt). Afterwards a scratch was done using a 10 µl white tip for each treatment. Then cells were washed with PBS and photographed using Leica DMI 6000 B microscope (Leica, Wetzlar, Germany). Cells were incubated for an additional 24 h after which the photographs were taken for the wounded area. The migrating cells were calculated according to the following formula:
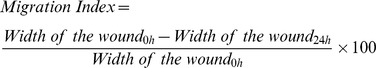



### Invasion assay

A total of 2.5×10^5^ cells/2 ml were plated in the upper chamber filter in serum free media. The cells were treated simultaneously with GSI (2.5 µM, 5 µM, 10 µM) and control (DMSO). The invasion assay utilized 6-well BD BioCoat™ Matrigel™ Invasion Chamber (BD Biosciences, Bedford, UK). These upper chamber filters were placed into the BD Falcon TC Companion Plate (BD Biosciences, Bedford, UK) containing 10% FCS. After 48 hrs incubation at 37°C, 5% CO_2_ atmosphere the cells on the upper surface of the membrane were mechanically removed with cotton swab. The invading cells were fixed in 100% ice-cold methanol (AppliChem, Darmstadt) and stained with 1% toluidine blue (Sigma-Aldrich, St. Louis, USA) in 1% borax (Sigma-Aldrich, St. Louis, USA). Cells were then counted under the microscope (Leica DM 5000 B, Wetzlar, Germany). The calculation of the invading cells was done according to the BD protocol:
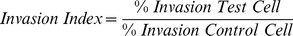



### Preparation of pancreatic tumor initiating CD44+/EpCAM+ cells for in vitro and in vivo analysis of tumorogenicity

CD44+/EpCAM+ populations were sorted from human pancreatic cancer cell line KP3. Cells were routinely sorted twice, and reanalyzed for purity (XDP, Beckman-Coulter). For *in vivo* experiments, 6×10^4^ sorted cells were resuspended in PBS/Matrigel (mixture 1∶1 volume, BD biosciences, San Diego) and were injected subcutaneously in to the left and right flank of 6-week-old female nude mice (*NMRI*-*nu/nu*). For *in vitro* the sorted cells were taken into cell culture and were treated for Western blots and cell proliferation assay with GSI inhibitor (Calbiochem, Darmstadt, Germany).

### Ethics Statement

The mice used in this study were maintained in the animal care of Medizinischen Hochschule Hannover (MHH) facility. All experimental protocols were reviewed and approved by institutional guidelines for animal care of MHH and lower Saxony (protocol no: AZ: 10/089), and all studies were performed according to the methods approved in the protocol.

### Animals

Female nude mice (*NMRI*-*nu/nu*) were purchased from Charles River Laboratories International (Wilmington, USA). Seven mice were treated with vehicle and eight with γ-Secretase inhibitor IX. Tumor appearance was inspected every alternate day by visual observation and caliper measurement. Treatment started when the tumor volume reached a size of 10 mm^3^. After 5 weeks of treatment, the mice were sacrificed; size of tumor was measured by caliper and tumors were harvested. Tumor volume was calculated using the following formula: Tumor volume V = [(ð\6)×(Length)×(Width^2^)]. The tumor tissues were snap frozen for protein analysis and fixed in 4% formalin for histology.

### Immunoblotting

Cells cultured with GSI treatment for immunoblots were scraped and rinsed with cold phosphate-buffered saline (PBS). The harvested cells were further lysed in lysis buffer containing 20 mM Tris, 150 mM NaCl, 1 mM EDTA, 1 mM EGTA, 1% Triton X-100 and protease and phosphatase inhibitor Protease Inhibitor Cocktail Tablets Roche, Mannheim). The concentrations of extracted proteins were determined using DC protein assay kit (Biorad, München) following manufacturer's instruction. The adsorption was measured at 650–750 nm with a microplate reader (Titertek-Berthold, Pforzheim). The protein concentrations were calculated via a standard curve. For immune blotting the cell lysates were loaded at the protein concentration of 100 µg per well. Gel electrophoreses (12% acrylamide gels) was run at 100 V.(Biorad, München). After the transfer to the PDVF membranes (PerkinElmer, Rodgau, Germany) at 100 V for 1 hr 20 minutes, the blots were blocked with 5% dried milk (AppliChem, Darmstadt, Germany) for 30 minutes at room temperature. These membranes were further probed with primary antibodies Hes1 (1∶10,000), E-Cadherin (1∶1000; Cell signaling, 24E10), N-Cadherin (2∶10,000; Millipore, EPR1792Y), Slug (1∶1000; Cell signaling, C19G7), Vimentin (1∶1000; Santa cruz, sc-7557), Actin (2∶10.000; Sigma, AC-74), CD44 (1∶1000; Cell signaling, 156-3C11) and Epcam (1∶1000; Cell signaling, VU1D9). The signal was detected by Amersham Hyperfilm ECL (GE Healthcare Limited, Buckinghamshire, UK).

### Histology

Sections were fixed in 4% formalin (Sigma-Aldrich, St. Louis, USA) overnight, stored in PBS and embedded in paraffin. For immunohistochemistry, slides were deparaffinized in rotihistol and rehydrated in ethanol (40%, 70%, 90% and 100% ethanol). For Ki-67 staining, Antigen retrieval was performed by heating the slides in pressure cooker with Antigen Unmasking Solution (Vector Laboratories, Inc., Burlingham) The slides were then washed in PBS and incubated for 10 min in 1% H_2_O_2_, rinsed with PBS, and incubated 1 h in blocking solution (5% normal serum þ 0.3% Triton X-100, Vector Laboratories, Inc., Burlingham). Hybridization with the primary antibody Ki-67 (1∶100; Novacastra,U.K.) was carried out overnight at 4°C. After PBS rinse, secondary antibody (1∶200; GE Healthcare, UK) was incubated for 1 h. The manufacturer's protocols were used for ABC and DAB substrates (Vector, Laboratories, Inc., Burlingham). Then, slides were counterstained with hematoxylin and dehydrated in 40%, 70%, 90%, and 100% ethanol. Finally, slides were cleared with Rhotihistol (Roth, Karlsruhe, Germany) and mounted with Permount Toluene Solution (Fisher Scientific, New Jersey, USA).

### Soft Agar Assay

The human PDAC cell line KP3 was used for colony formation assay. Soft agar plates were prepared in 60 mm plates with bottom layer of 1% nobel agar (Difco; BD Biosciences, Franklin Lakes, NJ) in RPMI 1640+L-Glutamine+25 mM HEPES (Invitrogen, Karlsruhe, Germany). 6×10^4^ cells/well were suspended in 3 mL of 0.5% of agarose along with the drug (GSI) and control (DMSO)) and were seeded as a top layer on to 1% agar coated plates. The cells were incubated for 2 weeks at 37°C in a humidified atmosphere containing 5% CO_2_ and counterstained with p-iodonitotetrazonium violet (Sigma). The number and size of colonies were determined after 2 weeks.

### Real-time PCR

RNA was purified from the treated and untreated KP3 and BxPC3 cells (Qiagen Rneasy Mini Kit, Hilden, USA). iScript cDNA Synthesis Kit (Biorad, USA) was used for the synthesis of cDNA with oligonucleotide primers (Thermo cycler Hielscher, Stahnsdorf). For quantitative SYBR green real-time PCR, sets of primers were obtained from Sigma-Aldrich: Hes1F- 5′GTGCATGAACGAGGTGACCC3′; Hes1R-5′GTATTAACGCCCTCGCACGT3′; GAPDH F-5′TTGATTTTGGAGGGATCTCG3′, GAPDH R-5′GAGTCAACGGATTTGGTCGT3′. Samples were run in triplicates at 5 µL of cDNA per well and detected using ABI Prism 7000 system, USA. Hes1 expression in cDNA samples were evaluated using SYBR-green labelling (Agilent, USA). The obtained results were normalized to GAPDH and relative quantification was assessed by comparative threshold-cycle method. mRNA gene expression is presented as fold increase calculated in relation to DMSO treated cells, after normalisation against GAPDH.

### Statistical Analysis

All the experiments were repeated 2–3 times. The results were analyzed using software Graphpad prism version 5.0 (GraphPad Software, San Diego, CA, USA) and SPSS Version 11.0 (SPSS, Chicago, USA). The tests include one way ANNOVA analysis of variance and student's *t*-test along with Bonferroni post test and paired and unpaired t-tests. Differences were considered as statistically significant when the P-value was less <0.05.

## Results

### Effect of γ-secretase inhibitor IX (GSI IX) on human pancreatic cancer cell line proliferation, apoptosis and colony formation

**Figure 1:**
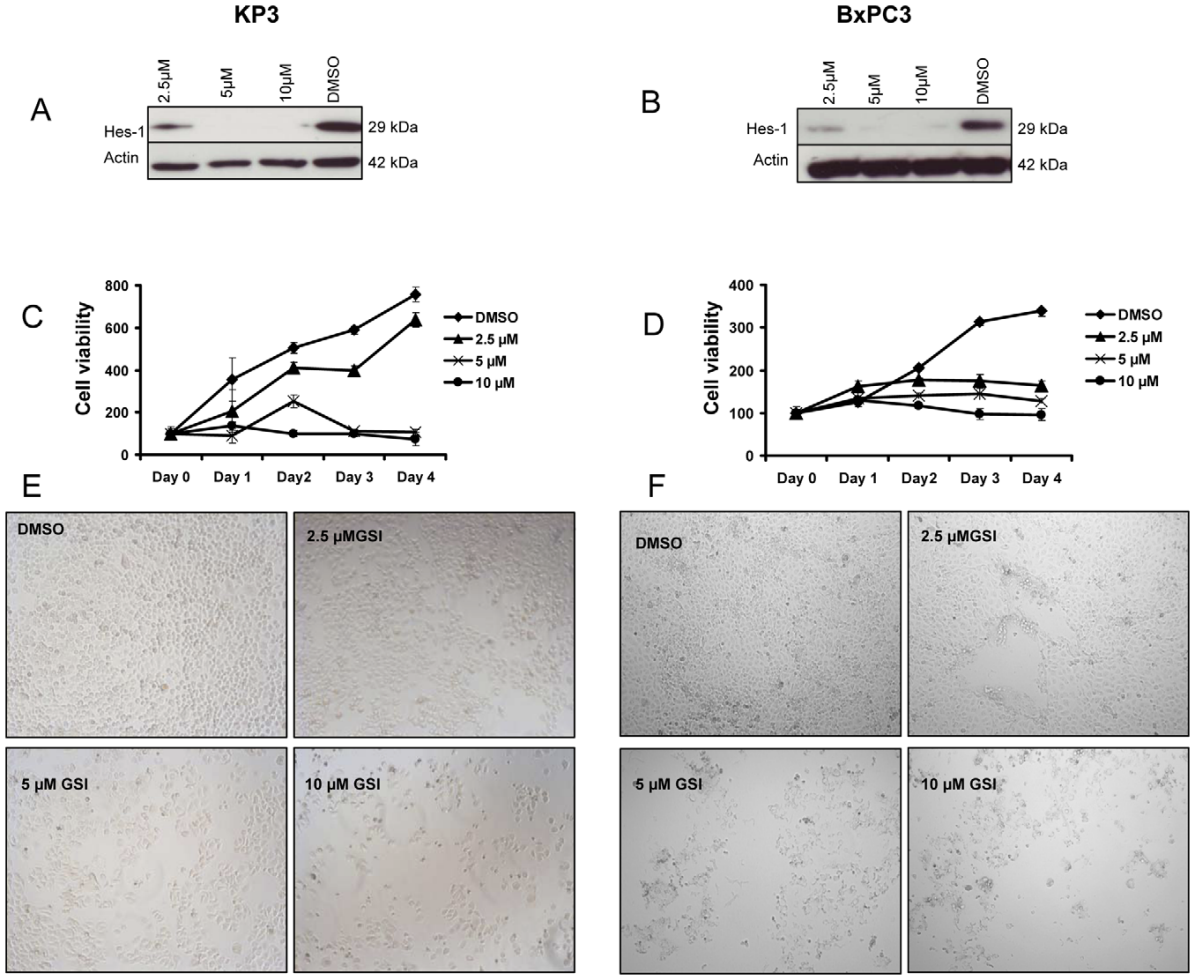
GSI IX inhibits cell proliferation in human pancreatic cancer cell lines and down regulates the Notch pathway downstream target Hes1. (A) KP3 and (B) BxPC3 cells were treated with GSI (2.5 µM, 5 µM, 10 µM) and control (DMSO) for 96 hrs showed a down regulation of Hes1 protein by Western Blot analysis. GSI treatment resulted in a shift in the growth curves. The cell proliferation of (C) KP3 and (D) BxPC3 was measured by cell proliferation assay, GSI inhibited cell proliferation in a dose- and time-dependent manner. Note that these results reveal the anti-proliferative effects of GSI on human pancreatic cancer cells. Light microscopic pictures (10× magnification) were taken at 96 h to show the effect of GSI on cell proliferation of (E) KP3 and (F) BxPC3.

Whereas a variety of Notch inhibitors have been tested on pancreatic cancer cell lines and shown to have cytostatic/cytotoxic effects, the specific cell biologic impact of this approach remains incompletely characterized. We first confirmed the sensitivity of the KP3 and BxPC3 pancreatic cancer cell lines with different tumorigenicity to Notch inhibition using GSI IX. Cell proliferation assays showed GSI IX treatment reduced the number of viable KP3 and BxPC3 cells in a dose and time dependent manner (Fig. 1C–F), consistent with prior studies of Notch inhibition in PDAC. As expected, Western blot and RT-PCR showed that GSI IX treatment strongly suppressed expression of the Notch downstream target Hes1 at 96 hrs, whereas expression was moderately decreased at 48 h time point (Fig. 1A–B, Fig. S1A–B, 2C–D). We found that GSI IX treatment induced significant levels of apoptosis in these pancreatic cancer cell lines, with levels increasing with drug dose (Fig. S3A–B). Finally, GSI IX strongly inhibited the ability of KP3 cells to form colonies in soft agar compared to DMSO treated cells (Fig. S2A–B). These studies suggest that Notch signaling is necessary for the proliferation, survival, and anchorage independent clonogenic growth of these pancreatic cancer cell lines.

### Targeting of Notch signalling results in an inhibition of human pancreatic cancer cell migration and invasion

**Figure 2:**
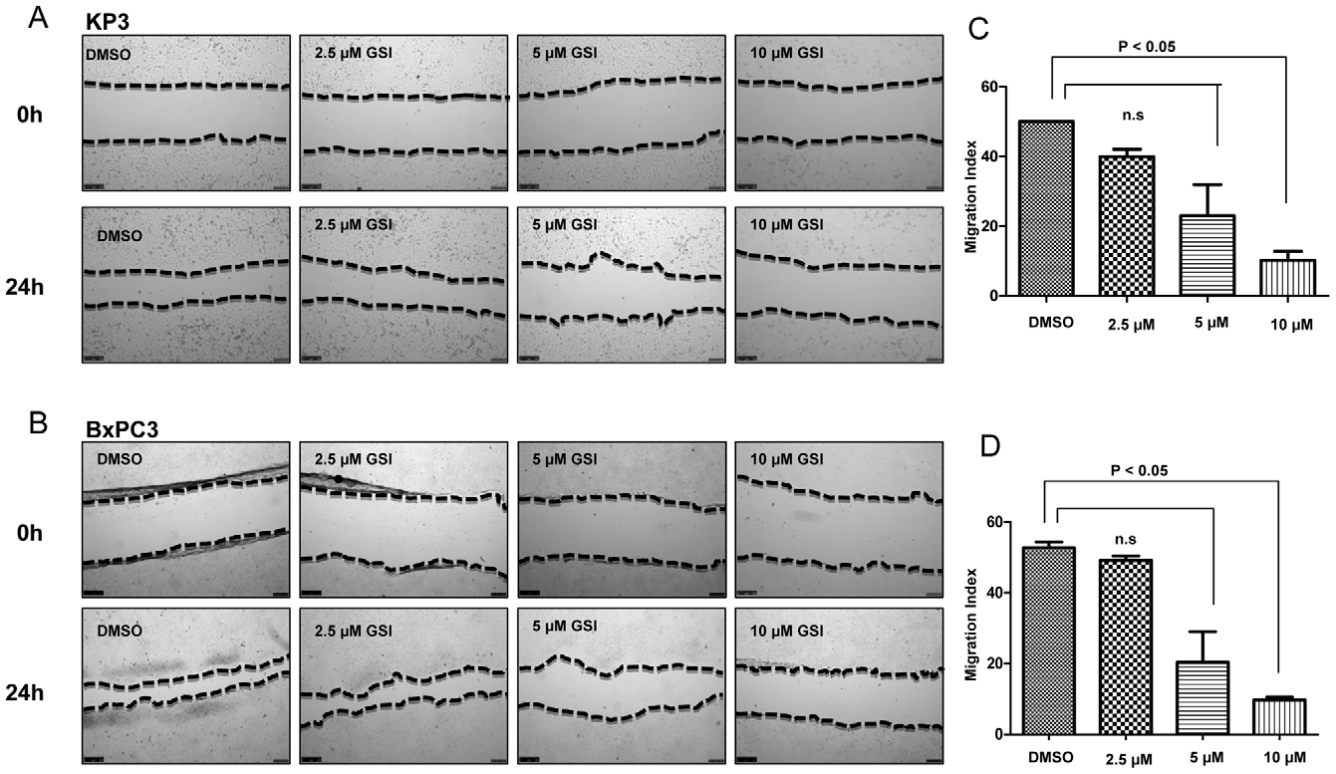
Notch plays a pivotal role for the regulation of migration in human pancreatic cancer cells. Treatment with GSI IX suppresses the migration potential of the human pancreatic cancer cell lines KP3 and BxPC3. Wound healing experiments of (A) KP3 and (B) BxPC3 cells cultured with GSI (2.5 µM, 5 µM, 10 µM) or control (DMSO). A scratch was made at (time 0 h) in both KP3 and BxPC3 and maintained for 24 h in conditioned medium with GSI or DMSO. The dotted lines are representing the edges of the wound. Photographs were taken under light microscope (10× magnification). After 24 h (A) Kp3 and (B) BxPC3 showed significant inhibition under 5 and 10 µM GSI treatment. In DMSO treated cells 80% to 90% of the wound healing was observed after 24 hrs. (C,D) The migration index was calculated as described in Material and Methods and plotted in bar graphs. P values were calculated with ANOVA analysis of variance along with Bonferroni post test. The error bar represents standard deviation. Differences were considered as statistically significant when the P-value was less <0.05 and non significant “n.s.” when the P-value was higher >0.05. The error bar represents standard deviation.

Notch signalling has been implicated in the invasive growth of a number of cancer types. Thus, we sought to explore the impact GSI IX on this process in human pancreatic cancer cell lines. First, we examined cell motility by employing wound healing assays in the presence or absence of GSI IX. Twenty four hours after the scratch, cell migration into the wound was captured under the microscope at 10× magnification (Fig. 2A–D). For both KP3 and BxPC3 cells, significant inhibition of wound closure was seen with treatment of 5–10 µM GSI IX (P<0.05). In contrast, 80–90% wound healing was seen after 24 h in all untreated cells. Thus, GSI can effectively inhibit the migration of pancreatic cancer cells.

**Figure 3:**
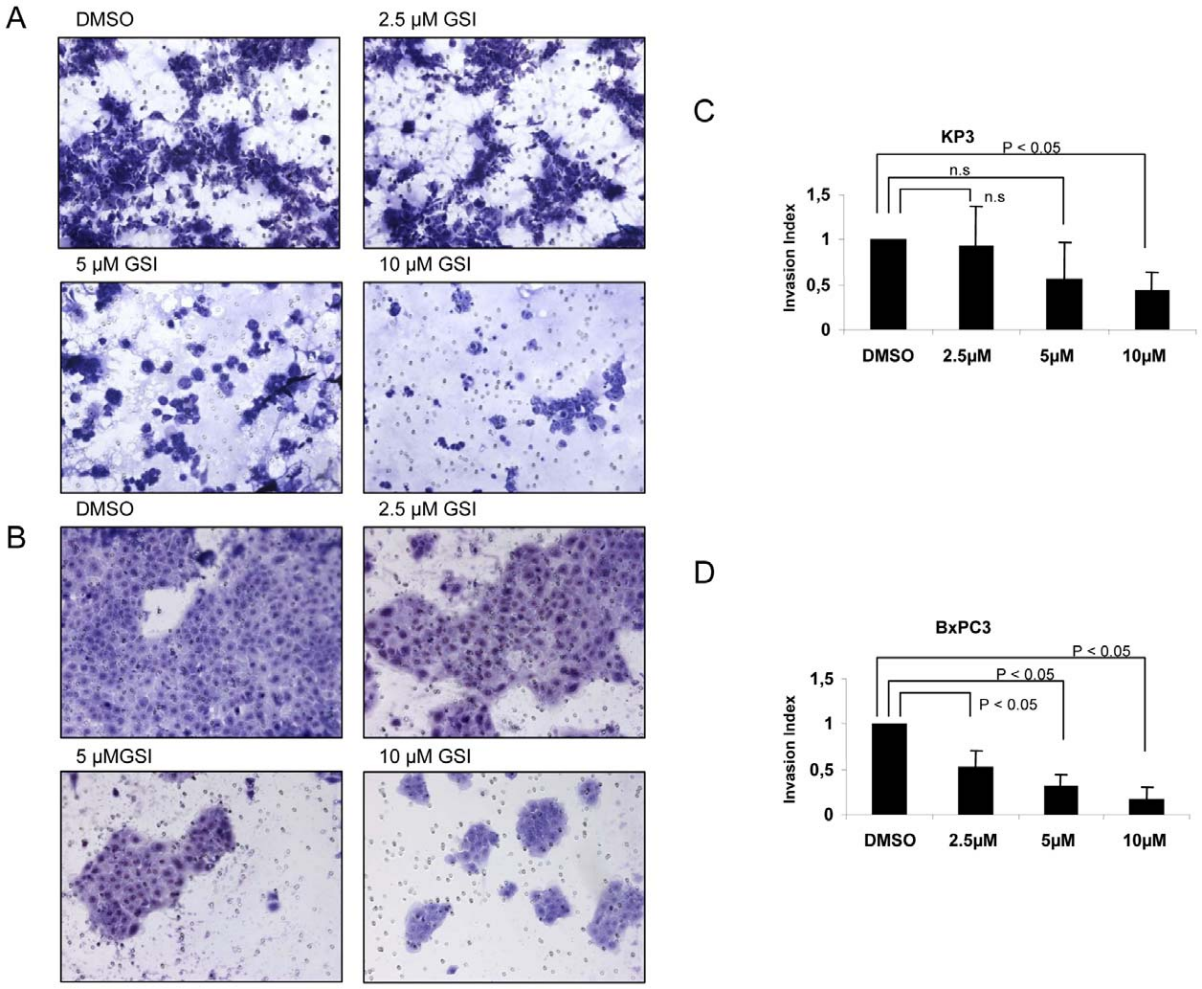
GSI IX attenuate invasion of human pancreatic cancer cells. KP3 and BxPC3 cell lines were treated for 48 h with control (DMSO) and GSI (2.5 µM, 5 µM, 10 µM) to investigate the effect of GSI on invasiveness of pancreatic cancer cell lines. The number of cells that invaded through the membrane was determined by light microscope (20× magnification) counterstained and invasion index was calculated as described in Material and Methods and plotted in bar graphs. Both (A) KP3 and (B) BxPC3 showed significant decrease in number of invading cells by light microscope. Note the slight difference of invasion index between (C) Kp3 and (D) BxPC3 cells. P values are calculated with ANOVA analysis of variance along with Bonferroni post test. The error bar represents standard deviation. Differences were considered as statistically significant when the P-value was less <0.05 and non significant “n.s.” when the P-value was higher >0.05. The error bar represents standard deviation.

Next, we tested cell invasion with transwell chambers. Pancreatic cancer cells were plated in wells of an invasion chamber in the presence of different drug concentrations and experiments were conducted as described in Material and Methods. As shown in Fig. 3A–D, the invasion of pancreatic cancer cells was significantly reduced (P<0.05) upon treatment with GSI IX, with reductions of up to 75%–80% in the number of invading cells compared to the control group. Thus, treatment with GSI IX has an anti-invasive effect on human pancreatic cancer cell lines.

### γ-secretase inhibitor IX (GSI IX) prevents mesenchymal transition of pancreatic cancer cell lines

**Figure 4:**
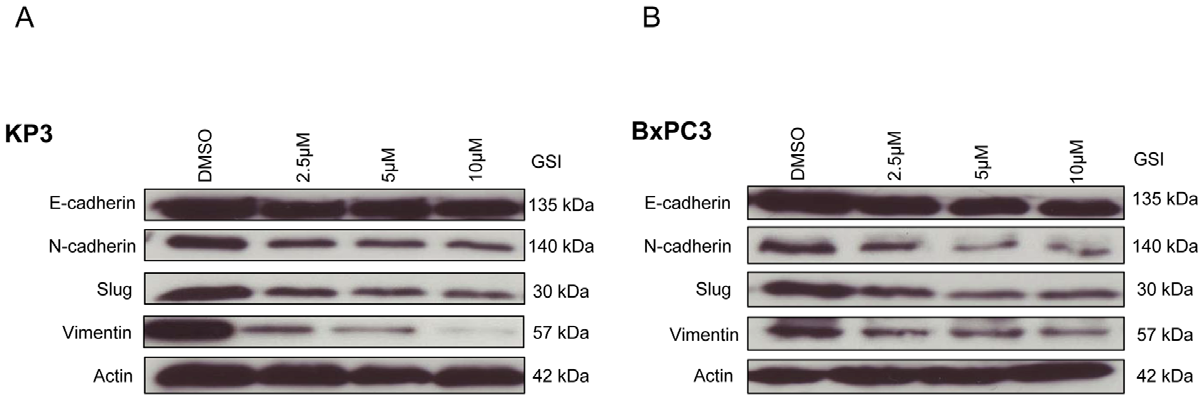
Change in the expression of epithelial and mesenchymal cell markers after GSI IX treatment in human pancreatic cancer. (A) KP3 and (B) BxPC3 cells were treated with control (DMSO) and GSI (2.5 µM, 5 µM and 10 µM) for 96 h. The expression of EMT markers: E-cadherin, N-cadherin, Slug and Vimentin were analyzed by Western blot. β-actin was used as a loading control. Both (A) Kp-3 and (B) BxPC3 showed no change in expression of epithelial marker E-cadherin but resulted in a GSI dose-independent down regulation of mesenchymal markers N-cadherin and Vimentin. We also detected a down regulation of the EMT transcriptional factor Slug after GSI treatment for both pancreatic cancer cell lines.

Activation of Notch signalling can contribute to the acquisition of EMT, a process associated with invasion and metastasis [39], [40]. In order to further examine whether GSI IX can attenuate EMT in human pancreatic cancer cell lines we treated KP3 and BxPC3 cells with different GSI concentrations (2.5 µM, 5 µM, 10 µM) for 48 and 96 h. Pharmacological treatment resulted in a dose-dependent decreased expression of mesenchymal markers like N-cadherin, Vimentin and transcriptional factor Slug as assessed by Western Blot (Fig. 4A–B). The epithelial cell marker E-cadherin had no change irrespective of the dosage in both the cell lines (Fig. 4A–B). The data from Western blot analysis also demonstrated that after 48 h GSI treatment the deregulation of EMT was minor compared to 96 h GSI treatment results (Fig. S1C–D). Therefore, these data show that GSI treatment could selectively inhibit the EMT phenotype in human pancreatic cancer cell lines.

### γ-secretase inhibitor IX (GSI) attenuates cell proliferation and mesenchymal program of sorted pancreatic tumor initiating CD44+/EpCAM+ cells

**Figure 5:**
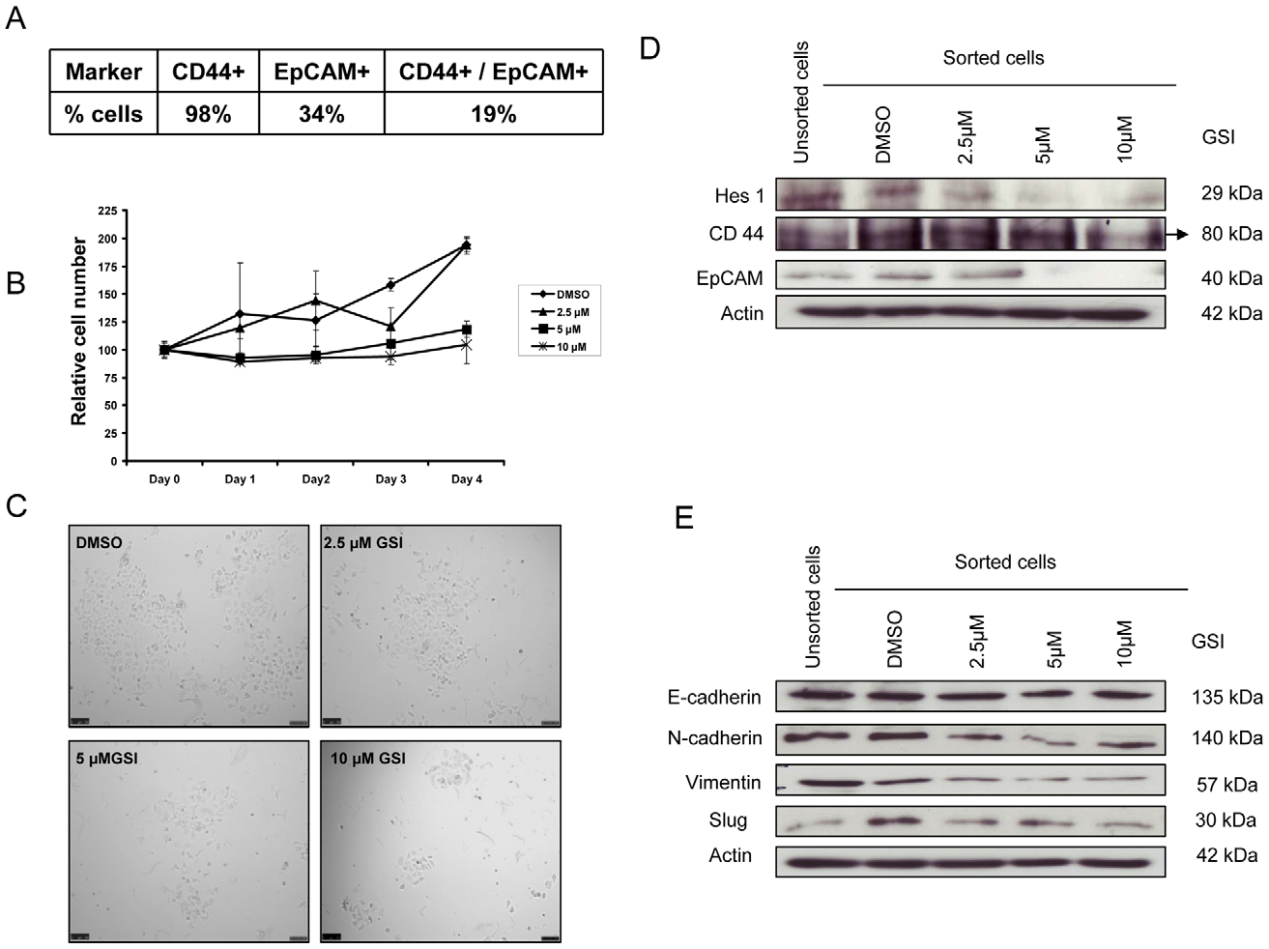
GSI treatment in sorted pancreatic tumor initiating CD44+/EpCAM+ cells reduces cell proliferation and selectively inhibits EMT. (A) Table showing the expression of CD44+, EpCAM+ cells and the combination of CD44+/EpCAM+ cells in pancreatic cancer cell line KP3. (B) CD44+/EpCAM+ cells were treated with GSI (2.5 µM, 5 µM and 10 µM) and control (DMSO) for 48 h to determine the role of Notch in regulating the cell proliferation. Cell proliferation was inhibited in a dose- and time-dependent manner. Note that these results reveal the anti-proliferative effects of GSI on human pancreatic tumor CD44+/EpCAM+ initiating cells. (C) Light microscopic pictures (10× magnification) were taken at 48 h to show the effect of GSI on cell proliferation. (D) The down regulation of the Notch pathway was confirmed by Western Blot for Notch downstream target Hes1. Compared to unsorted and sorted DMSO treated cells Hes1 showed a dose-dependent down regulation after GSI treatment. (D) CD44 and EpCAM were down regulated in a dose dependent manner. The black arrow is marking the protein lane of CD44. (E) Epithelial marker E-cadherin was unaltered, but mesenchymal marker N-cadherin, Vimentin and Slug showed dose-dependent down regulation.

It has been shown in many studies that Notch signaling plays a role in the stem cell renewal and cell fate determination in neural, hematopoietic as well as in embryonic stem cells [41]. In PDAC, it is reported that CSCs are highly tumorigenic and contain high levels of Notch1 and Notch2 [16], [37]. We hypothesized that the deregulation of the Notch signalling pathway by GSI could be a useful approach to treat human pancreatic cancer. The pancreatic cancer cell line KP3 expressed 98% CD44 positive and 34% of EpCAM positive cells, but when the markers were sorted together only 19% of cells were positive for both of the markers (Fig. 5A). To further determine the effect of GSI on sorted tumor initiating CD44+/EpCAM+ cells, we analyzed the effect of GSI by cell proliferation assay. As shown in Figure 5B–C GSI, inhibited the cell proliferation in a dose and time dependent manner. These data reveal the anti-proliferative effects of GSI on tumor initiating CD44+/EpCAM+ cells. We also performed apoptotic assay in these sorted tumor initiating CD44+/EpCAM+ cells in order to study the mechanism by which GSI IX treatment affects cell growth. The results indicated that GSI IX treatment induced significant levels of apoptosis at 10 µM concentration (Fig. S4). Furthermore we studied the effect of GSI treatment on Notch downstream target Hes1 and tumor initiating cell markers CD44 and EpCAM in sorted CD44+/EpCAM+ cells. The results from the Western Blots showed a down regulation of Hes1, CD44 and EpCAM in a dose dependent manner (Fig. 5D). These data suggest that down regulation of Notch may thus contribute to the inhibition of tumor initiating cells or CSC.

We further observed the effect of GSI on regulation of EMT process in sorted CD44+/EpCAM+ cells. E-cadherin expression was stable and had no change independent of GSI dosage (Fig. 5E). Next we analyzed mesenchymal markers N-cadherin, Vimentin and Slug where we found that they are also down regulated in dose dependent manner (Fig. 5E). These data suggest that GSI inhibition can be a promising therapeutic intervention to eliminate pancreatic tumor initiating CD44+/EpCAM+ cells and partially prevent EMT phenotype.

### Intraperitoneal treatment with γ-secretase inhibitor IX (GSI) effectively inhibits the growth of CD44+/EpCAM+ xenograft tumors and targets EMT

**Figure 6:**
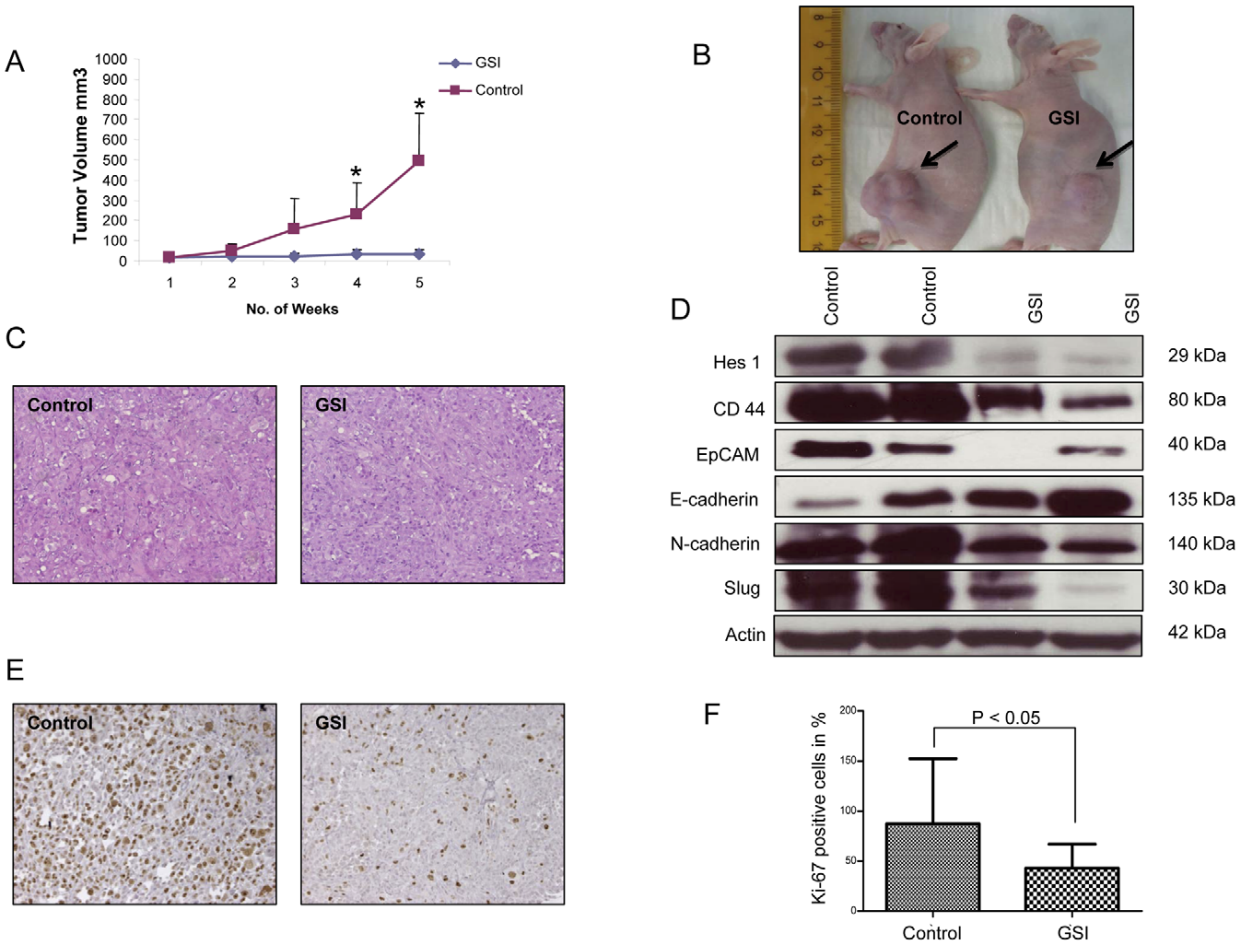
GSI IX inhibits the growth of subcutaneously injected pancreatic CD44+/EpCAM+ cells. (A) Diagram shows time course of tumor growth in GSI and vehicle treated animals. The tumor growth volume was significantly decreased in the treated compared to the control group. P values were calculated with student's *t*-test; (‘*’ signifies P<0.05) error bars represent SD. (B) Pictures of mice taken at 5 weeks. The left mouse is treated with vehicle, the right mouse with GSI. The arrows are marking the growing xenografts. (C) Histology images of representative xenograft tumors from vehicle and GSI treated mice (20× magnification). (E) Ki67 staining of vehicle and GSI treated xenografts. (F) Quantification of Ki67 staining in the xenograft tumors, showing significant reduction in Ki67+ cells following GSI treatment (20× magnification). Differences were considered as statistically significant when the P-value was less <0.05 and non significant “n.s.” when the P-value was higer >0.05. Error bars show standard deviation. (D) Western Blot analysis showed a down regulation of the Notch downstream target Hes1 in xenograft tumors of GSI treated animals compared to control mice. (D) Protein expression of CD44, EpCAM, E-cadherin, N-cadherin and Slug were analyzed by the Western blot analysis and β-actin was used as a loading control. Note that *in vivo* treatment with GSI is sufficient to reverse the EMT-associated “cadherin-switch” in xenograft tumors.

Given the ability of GSI to inhibit pancreatic tumor initiating CD44+/EpCAM+ cells *in vitro*, we further tested the *in vivo* role of GSI in a Xenograft nude mouse (*NMRI*-*nu/nu*) model. Sorted CD44+/EpCAM+ cells were subcutaneously injected into the left and right flank. Intraperitoneal treatment with either GSI or vehicle started when the tumor volume reached a size of 10 mm^3^. All mice were treated for 5 weeks, using an established 3 days on and 4 days off intermittent dose schedule [29].Xenograft tumors grew continuously in vehicle-treated animals, whereas GSI treatment significantly inhibited tumor growth (Fig. 6A–B). Histological analysis of explanted xenograft tumors from GSI or vehicle treated mice did not show any morphological differences (Fig. 6C).

To analyze the effect of GSI on proliferation of pancreatic CD44+/EpCAM+ cells in xenograft tumors we performed Ki67 staining of xenograft tumor tissues (Fig. 6E). The average proliferation rate was significantly decreased (P<0.05) in mice treated with GSI (Fig. 6F). We next confirmed by Western Blot that the Notch signaling pathway target gene Hes1 is down regulated in GSI treated Xenograft tumors (Figure 6D). We assessed the role of Notch for the maintenance of tumor initiating pancreatic CD44+/EpCAM+ cells and found that with down regulation of the Notch signalling pathway the expression of CD44 and EpCAM on protein level were dramatically decreased (Fig. 6D). To further delineate the role of Notch signaling in the regulation of EMT process in xenograft tumors, we analyzed some EMT markers. In contrast to our *in vitro* findings, the epithelial marker E-cadherin showed an induced expression in the GSI treated compared to the control group (Fig. 6D). Slug which is described as a transcriptional repressor of E-cadherin showed a down regulation under GSI treatment (Fig. 6D). The mesenchymal marker N-cadherin was also down regulated in the GSI group (Fig. 6D). Differently to the *in vitro* results, Vimentin was not expressed in either control or GSI treated xenograft tumors. These data validate that treatment with GSI causes a significant inhibition of pancreatic CD44+/EpCAM+ cells *in vivo*. It also shows that GSI has an antiproliferative effect on xenograft tumors and can influence the EMT process.

## Discussion

Notch inhibition has been regarded as an effective therapeutic strategy in many cancer studies. In the present work, we have used the γ-secretase inhibitor IX (GSI) to show that the Notch signalling pathway contributes to the acquisition of epithelial mesenchymal transition (EMT) and is associated with the maintenance of pancreatic tumor initiating CD44+/EpCAM+ cells. In accordance with our previous findings we have shown that treatment with GSI induced dose- and time-dependent growth inhibition in human pancreatic cancer cell lines, as indicated by cell proliferation assay. Importantly, in our experiments we used human pancreatic cancer cell lines from different origins: BxPC3 is a common available human pancreatic cancer cell line, cultured from a primary tumor without evidence of metastasis [42], [43], in contrast KP3 is obtained from liver metastases of a human pancreatic tumor [44]. We hypothesised that pancreatic cancer cell lines with different tumorigenicity profile might behave differently under GSI treatment.

Previously it was demonstrated that the Notch signalling pathway is specifically required for PDAC initiation and the chemical inhibition of Notch activation represses PDAC development [24]–[35]. In agreement with this data we found that expression of the Notch target gene Hes1 was significantly decreased when treated with GSI. Furthermore, we showed that GSI induced apoptotic cell death in a dose- and time-dependent manner. Additionally, GSI treated human pancreatic cancer cells had a greatly reduced capacity to form colonies.

Epithelial-to-mesenchymal transition (EMT) is the collection of events that allows the conversion of adherent epithelial cells into independent fibroblastic cells possessing migratory properties and the ability to invade the extracellular matrix [10]. Previous work has shown that the activation of Notch signalling contributes to the acquisition of EMT [39]. Furthermore, it is known that reduction of E-cadherin expression is associated with advanced PDAC stage and positive lymph nodes [45], [46] It was also demonstrated that activation of Notch 2 mediates an EMT phenotype [47] and that Notch 2 deficiency caused a phenotypical switch with EMT [34]. The high metastatic potential of pancreatic cancer underscores the importance to further investigate and inhibit migration and invasion. Indeed, we found that treatment with GSI resulted in an *in vitro* inhibition of migration and invasion using wound-healing assay and modified boyden chamber. We also showed a time- and dose-dependent decrease of mesenchymal markers like N-cadherin, Vimentin and of the transcriptional factor Slug. On the other hand, the epithelial marker E-cadherin was unchanged, this can be explained by the fact that both of the cell lines investigated are epithelial and remain epithelial upon the treatment with GSI. It is known that Notch directly up regulates Slug in endothelial cells and expression of this transcriptional factor is required for repression of Notch mediated vascular endothelial cadherin promoter as well for promoting migration of transformed endothelial cells [48]. These mesenchymal markers are known to be strongly required for pancreatic cancer carcinogenesis and can be successfully altered by GSI application. Taken together, these results suggest that treatment with GSI selectively inhibits EMT. Notably, for these experiments we did not observe any significant differences between KP3 and BxPC3 pancreatic cancer cell lines indicating that GSI IX can block human pancreatic cancer cell lines independent of metastasis background.

Consistent to its role in other solid tumors, CSCs are also responsible for tumor recurrence as well as tumour metastasis in pancreatic cancer. Several studies have shown the significant role of human pancreatic CD44+, CD133+ and ESA+ (EpCAM+) CSCs [40]. Hermann et al. has reported that human pancreatic CSCs are highly tumorigenic and others have shown that CSCs contain high levels of Notch1 and Notch2 [16], [37]. We hypothesized that deregulation of the Notch signalling pathway by GSI will be a useful approach to treat pancreatic cancer stem cells. Li et al. also showed successfully that injection of human PDAC CD44+/ESA+ (EpCAM+) cells developed xenograft tumors, whereas CD44−/ESA− (EpCAM−) cells had a significant decrease in tumorigenic potential [23]. Besides that it was demonstrated that CD44+ cells are responsible for gemcitabine resistance in PDAC cells [49]. Based on this prior work, we have chosen to analyze pancreatic tumor initiating CD44+/EpCAM+ cells. In contrast, we did not use xenografts from human PDAC, but rather analyzed the common used human metastatic pancreatic cancer cell line KP3.

Our *in vitro* results clearly showed that the inhibition of Notch signalling by GSI resulted in a dose-dependent growth attenuation of human pancreatic tumor initiating CD44+/EpCAM+ cells, down regulated the Notch target Hes1 and decreased EMT-related molecules. This might confirm the role of Notch in the maintenance of the CD44+/EpCAM+ subpopulation. We further proceeded to test the efficacy of GSI on pancreatic tumor initiating CD44+/EpCAM+ cells. In pancreatic cancer xenografts, we found a significant inhibition of tumor growth in all of the GSI treated xenografts compared to the vehicle group. To attenuate possible drug side effects, we followed an established 3-day on and 4-day off dosing schedule [29]. GSI and vehicle treated animals showed no significant changes of body weight or other abnormalities. Our current study clearly demonstrated that *in vivo* inhibition of pancreatic tumor initiating CD44+/EpCAM+ cells by GSI resulted in a down regulation of mesenchymal cell markers and up-regulation of epithelial cell markers and can suppress tumorigenesis.

Taking into account other signaling pathways like Hedgehog and mTOR which are known to affect pancreatic CSCs as well, we cannot rule out by our analysis that GSI IX also has some other indirect effects.

Our findings support the previously identified CD44+ and EpCAM+ CSC population in human PDAC. In conclusion, our data confirmed and expanded on the CSC hypothesis and also showed an evidence for the first time that CD44+ and EpCAM+ xenografts underwent EMT partly upon the treatment with GSI. Taken together, this study demonstrates the central role of Notch signalling pathway in pancreatic cancer pathogenesis independent of their tumorigenicity and that pancreatic tumor initiating CD44+/EpCAM+ cells are down regulated by GSI. Future clinical investigations are needed to confirm these results and to establish GSI as a part of the treatment regime for pancreatic cancer patients.

## Supporting Information

Figure S1 **Expression of Hes 1 and epithelial and mesenchymal cell markers after 48 h of GSI IX treatment in human pancreatic cancer.** (A) The pancreatic cancer cell line KP3 treated with GSI (2.5 µM, 5 µM, 10 µM) and DMSO (control) for 48 h showed no change in Hes1 protein by Western Blot analysis. (B) The pancreatic cancer cell line BxPC3 treated with GSI (2.5 µM, 5 µM, 10 µM) and DMSO for 48 h showed a slight Hes1 decrease after same treatment regime compared to control. Expression of EMT markers (C) KP3 and (D) BxPC3 cells showed no change in expression of epithelial E-cadherin, N-cadherin and Slug, but resulted in GSI dose-dependent down regulation of Vimentin. Protein expression was analyzed by Western blot and β-actin was used as a loading control.
(TIF)

Figure S2 **GSI IX treatment significantly inhibited the colony formation ability and down regulates the Notch pathway downstream target Hes1.** (A) Soft agar assay of GSI-treated human pancreatic cancer cell line (KP3), with quantification on right (B). Compared to DMSO (control) GSI inhibits colony formation at a concentration of 10 µM. Note the significant difference in the number of colonies in KP3 cells treated with GSI. (C–D) RT-PCR Analysis of Notch signaling target Hes1 in KP3 and BxPC3 cell lines treated with GSI for 96 hrs. (C) KP3 showed significant decrease in Hes1 expression in all the treatments when compared to the control. (D) BxPC3 showed a minor down regulation of Hes1. P values are calculated with ANOVA analysis of variance and student's *t*-test along with Bonferroni post test. The error bar represents standard deviation. Differences were considered as statistically significant when the P-value was less <0.05 and non significant “n.s.” when the P-value was higher >0.05.
(TIF)

Figure S3 **GSI IX induces dose-dependent apoptosis in human pancreatic cancer cell lines.** KP3 and BxPC3 cells were treated with control (DMSO) and GSI (2.5 µM, 5 µM, 10 µM) for 96 hrs and apoptosis was quantified by staining with Annexin V and propidium iodide (PI) using flow cytometry. Both (A) Kp3 and (B) BxPC3 showed significant increase in apoptotic cells in the highest dose (10 µM) when compared to the control. P values are calculated with ANOVA analysis of variance along with Bonferroni post test. The error bar represents standard deviation. Differences were considered as statistically significant when the P-value was less <0.05 and non significant “n.s.” when the P-value was higher >0.05. The error bar represents standard deviation.
(TIF)

Figure S4 **GSI IX induces dose-dependent apoptosis in sorted CD44+/EpCAM+ cells.** Sorted CD44+/EpCAM+ cells were treated with control (DMSO) and GSI (2.5 µM, 5 µM, 10 µM) for 48 hrs and apoptosis was quantified by staining with Annexin V and propidium iodide (PI) using flow cytometry. Results showed significant increase in apoptotic cells in the highest dose (10 µM) when compared to the control. P values are calculated with ANOVA analysis of variance along with Bonferroni post test. The error bar represents standard deviation. Differences were considered as statistically significant when the P-value was less <0.05 and non significant “n.s.” when the P-value was higher >0.05. The error bar represents standard deviation.
(TIF)
