# Supplementary figures and images for: Epithelial Mesenchymal Transition and Pancreatic Tumor Initiating CD44+/EpCAM+ Cells Are Inhibited by γ-Secretase Inhibitor IX
Source: PLoS One. 2012 Oct 19;7(10):e46514. doi: 10.1371/journal.pone.0046514 (PMC3477166; doi:10.1371/journal.pone.0046514)

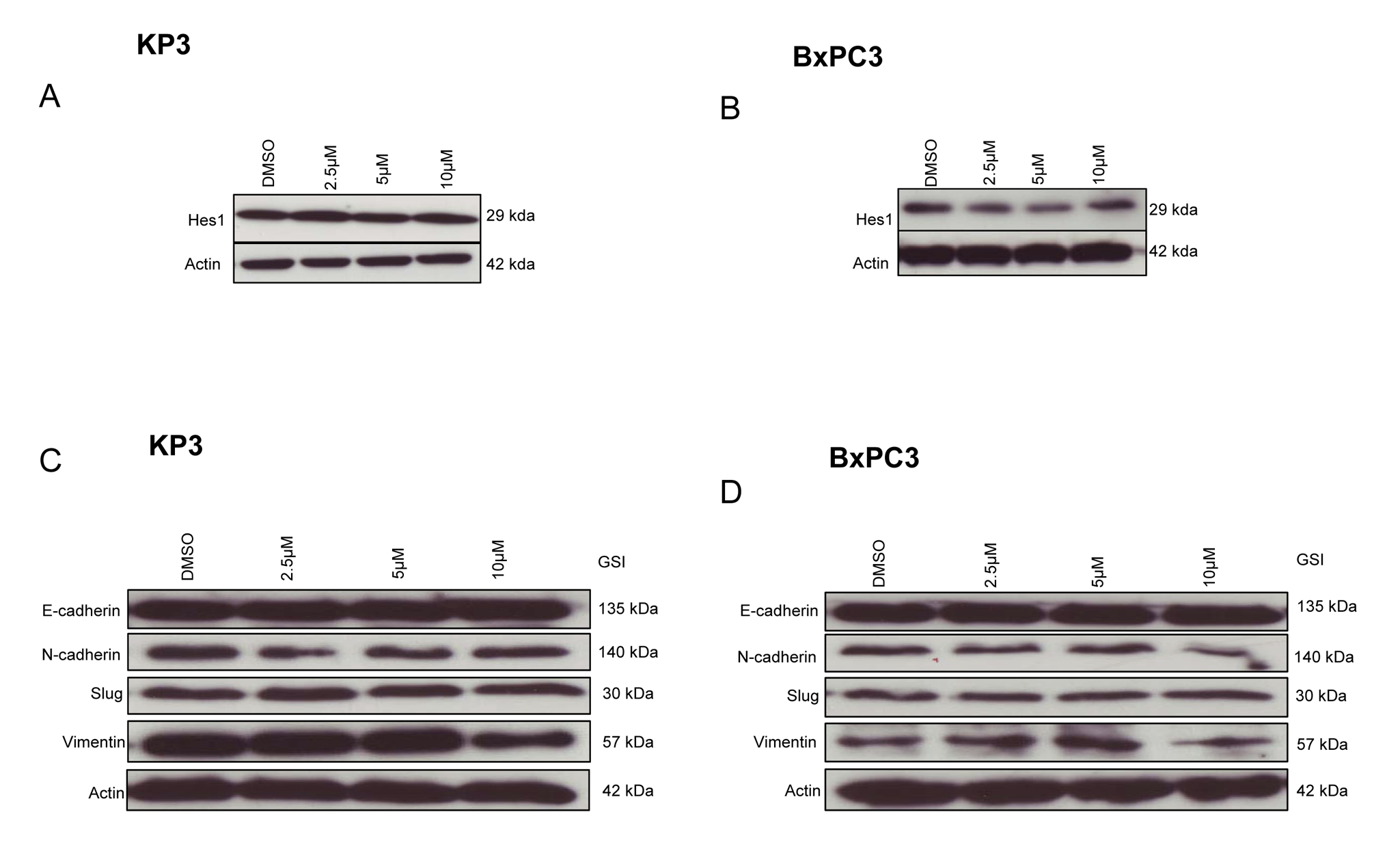

Supplement: Figure S1 — Expression of Hes 1 and epithelial and mesenchymal cell markers after 48 h of GSI IX treatment in human pancreatic cancer. (A) The pancreatic cancer cell line KP3 treated with GSI (2.5 µM, 5 µM, 10 µM) and DMSO (control) for 48 h showed no change in Hes1 protein by Western Blot analysis. (B) The pancreatic cancer cell line BxPC3 treated with GSI (2.5 µM, 5 µM, 10 µM) and DMSO for 48 h showed a slight Hes1 decrease after same treatment regime compared to control. Expression of EMT markers (C) KP3 and (D) BxPC3 cells showed no change in expression of epithelial E-cadherin, N-cadherin and Slug, but resulted in GSI dose-dependent down regulation of Vimentin. Protein expression was analyzed by Western blot and β-actin was used as a loading control. (TIF) [file pone.0046514.s001.tif]

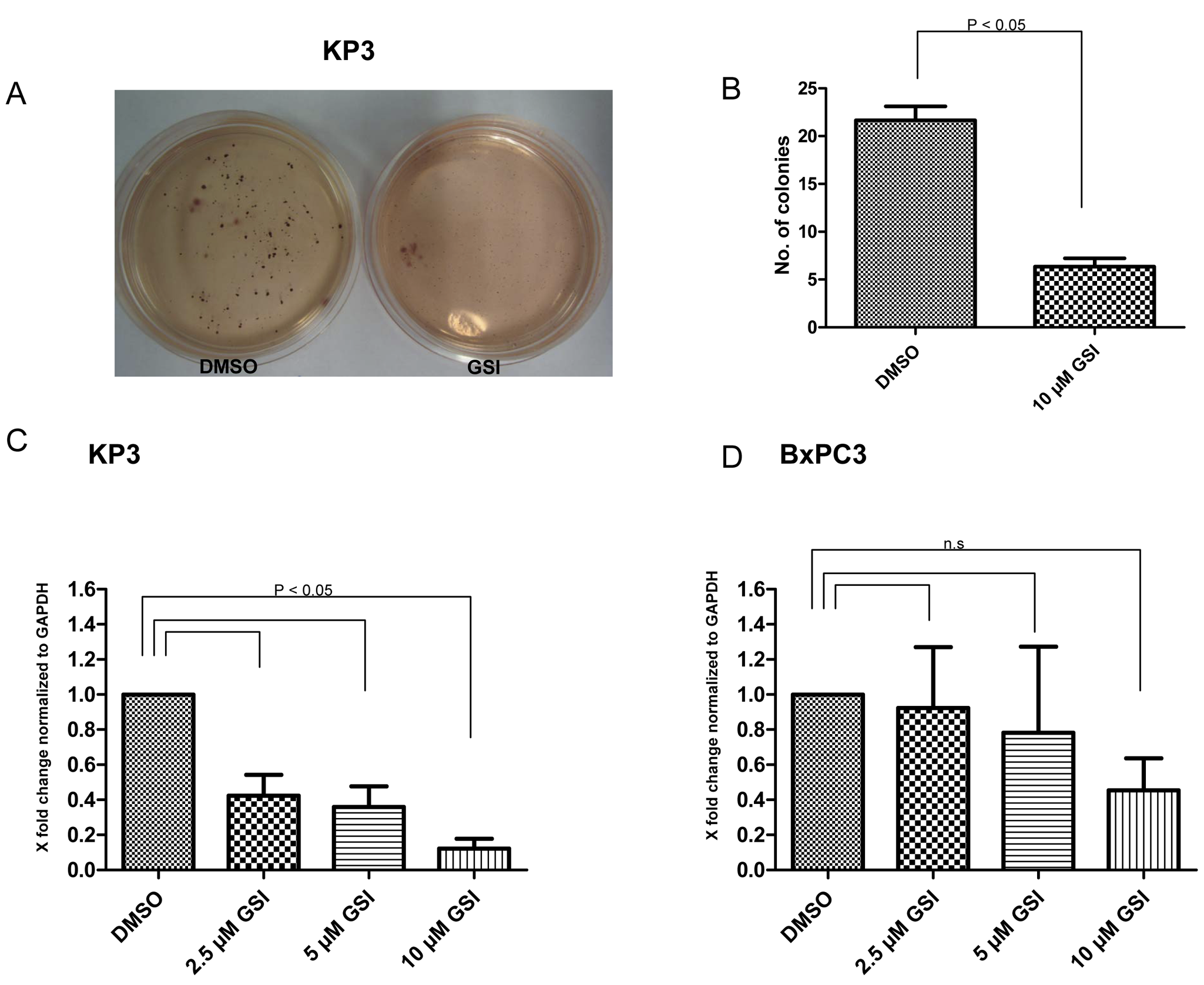

Supplement: Figure S2 — GSI IX treatment significantly inhibited the colony formation ability and down regulates the Notch pathway downstream target Hes1. (A) Soft agar assay of GSI-treated human pancreatic cancer cell line (KP3), with quantification on right (B). Compared to DMSO (control) GSI inhibits colony formation at a concentration of 10 µM. Note the significant difference in the number of colonies in KP3 cells treated with GSI. (C–D) RT-PCR Analysis of Notch signaling target Hes1 in KP3 and BxPC3 cell lines treated with GSI for 96 hrs. (C) KP3 showed significant decrease in Hes1 expression in all the treatments when compared to the control. (D) BxPC3 showed a minor down regulation of Hes1. P values are calculated with ANOVA analysis of variance and student's t-test along with Bonferroni post test. The error bar represents standard deviation. Differences were considered as statistically significant when the P-value was less <0.05 and non significant “n.s.” when the P-value was higher >0.05. (TIF) [file pone.0046514.s002.tif]

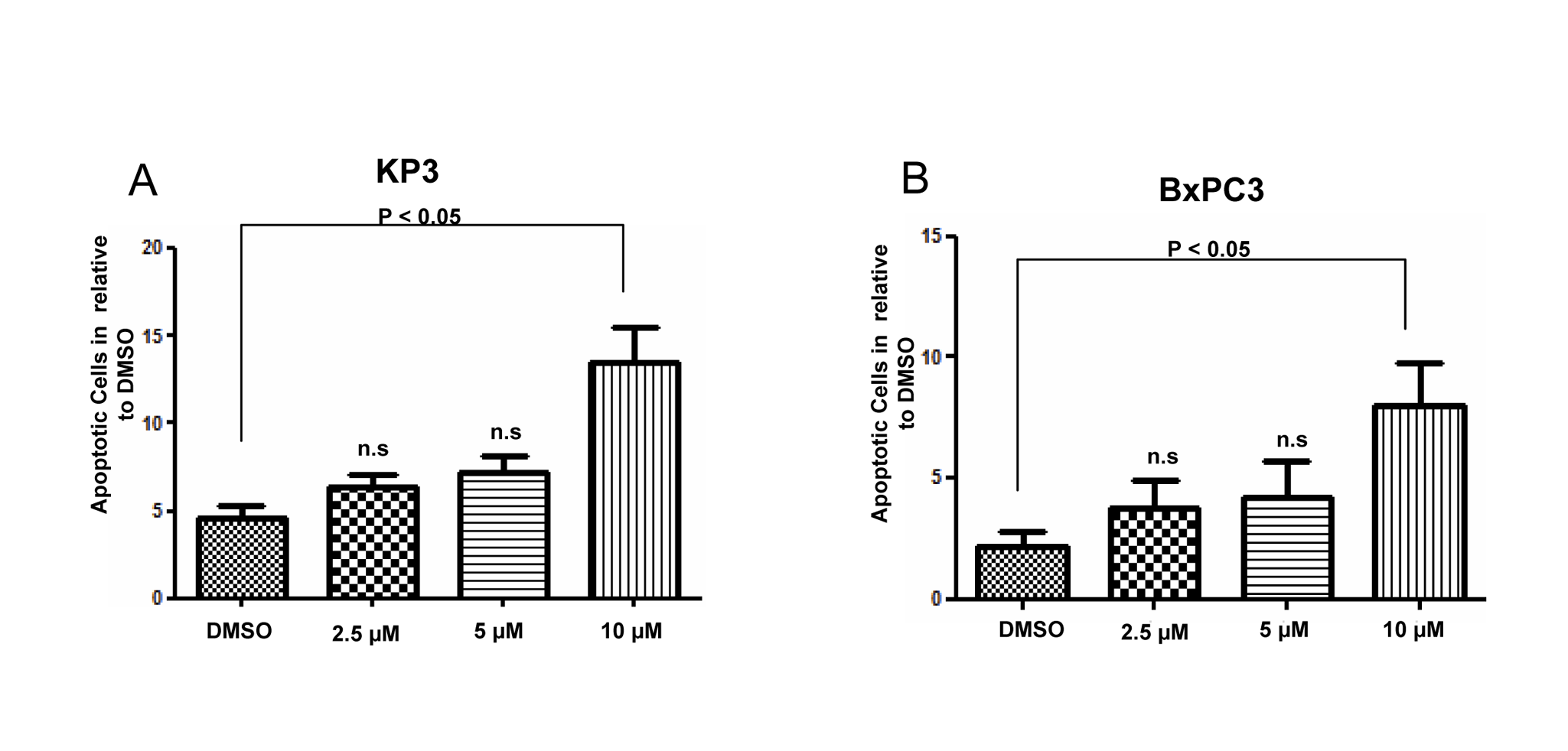

Supplement: Figure S3 — GSI IX induces dose-dependent apoptosis in human pancreatic cancer cell lines. KP3 and BxPC3 cells were treated with control (DMSO) and GSI (2.5 µM, 5 µM, 10 µM) for 96 hrs and apoptosis was quantified by staining with Annexin V and propidium iodide (PI) using flow cytometry. Both (A) Kp3 and (B) BxPC3 showed significant increase in apoptotic cells in the highest dose (10 µM) when compared to the control. P values are calculated with ANOVA analysis of variance along with Bonferroni post test. The error bar represents standard deviation. Differences were considered as statistically significant when the P-value was less <0.05 and non significant “n.s.” when the P-value was higher >0.05. The error bar represents standard deviation. (TIF) [file pone.0046514.s003.tif]

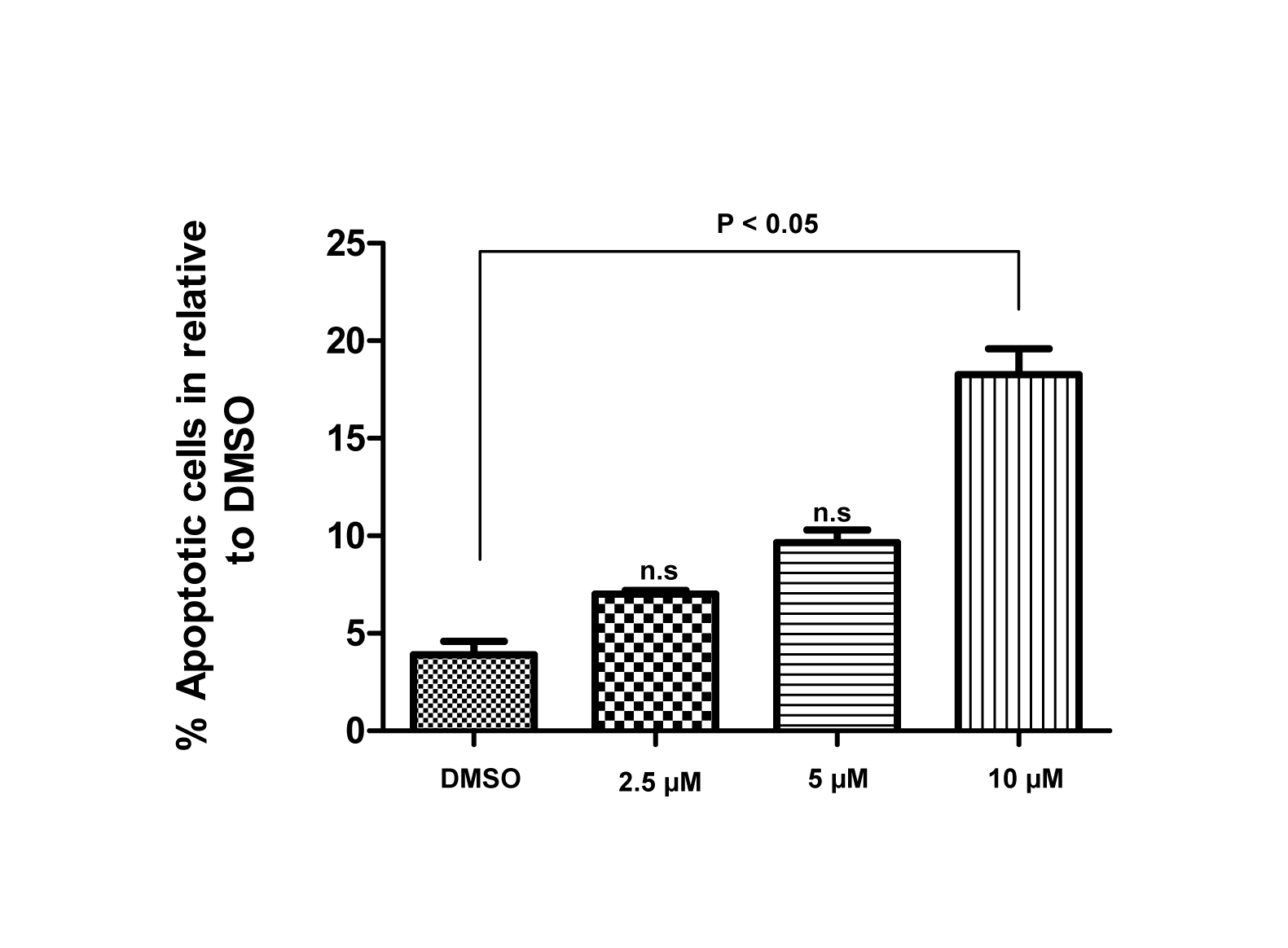

Supplement: Figure S4 — GSI IX induces dose-dependent apoptosis in sorted CD44+/EpCAM+ cells. Sorted CD44+/EpCAM+ cells were treated with control (DMSO) and GSI (2.5 µM, 5 µM, 10 µM) for 48 hrs and apoptosis was quantified by staining with Annexin V and propidium iodide (PI) using flow cytometry. Results showed significant increase in apoptotic cells in the highest dose (10 µM) when compared to the control. P values are calculated with ANOVA analysis of variance along with Bonferroni post test. The error bar represents standard deviation. Differences were considered as statistically significant when the P-value was less <0.05 and non significant “n.s.” when the P-value was higher >0.05. The error bar represents standard deviation. (TIF) [file pone.0046514.s004.tif]
